# Phenolic Bioactives as Antiplatelet Aggregation Factors: The Pivotal Ingredients in Maintaining Cardiovascular Health

**DOI:** 10.1155/2021/2195902

**Published:** 2021-08-17

**Authors:** Javad Sharifi-Rad, Cristina Quispe, Wissam Zam, Manoj Kumar, Susana M. Cardoso, Olivia R. Pereira, Adedayo O. Ademiluyi, Oluwakemi Adeleke, Ana Catarina Moreira, Jelena Živković, Felipe Noriega, Seyed Abdulmajid Ayatollahi, Farzad Kobarfard, Mehrdad Faizi, Miquel Martorell, Natália Cruz-Martins, Monica Butnariu, Iulia Cristina Bagiu, Radu Vasile Bagiu, Mohammed M. Alshehri, William C. Cho

**Affiliations:** ^1^Phytochemistry Research Center, Shahid Beheshti University of Medical Sciences, Tehran, Iran; ^2^Facultad de Ciencias de la Salud, Universidad Arturo Prat, Avda. Arturo Prat 2120, Iquique 1110939, Chile; ^3^Department of Analytical and Food Chemistry, Faculty of Pharmacy, Al-Andalus University for Medical Sciences, Tartous, Syria; ^4^Chemical and Biochemical Processing Division, ICAR-Central Institute for Research on Cotton Technology, Mumbai 400019, India; ^5^LAQV-REQUIMTE, Department of Chemistry, University of Aveiro, 3810-193 Aveiro, Portugal; ^6^Centro de Investigação de Montanha (CIMO), Instituto Politécnico de Bragança, Campus de Santa Apolónia, 5300-253 Bragança, Portugal; ^7^Functional Foods and Nutraceuticals Unit, Department of Biochemistry, Federal University of Technology, Akure, Nigeria; ^8^Department of Science Laboratory Technology, Ekiti State University, Ado-Ekiti, Nigeria; ^9^Pulmonology Department, Hospital Garcia de Orta, EPE, Almada, Lisboa, Portugal; ^10^Institute for Medicinal Plants Research “Dr. Josif Pančić”, Tadeuša Košćuška 1, 11000 Belgrade, Serbia; ^11^Department of Plant Production, Faculty of Agronomy, Universidad de Concepción, Chillan 4070386, Chile; ^12^H.E.J. Research Institute of Chemistry, International Center for Chemical and Biological Sciences, University of Karachi, Karachi, Pakistan; ^13^Department of Pharmacognosy and Biotechnology, School of Pharmacy, Shahid Beheshti University of Medical Sciences, Tehran, Iran; ^14^Department of Medicinal Chemistry, School of Pharmacy, Shahid Beheshti University of Medical Sciences, Tehran, Iran; ^15^Department of Pharmacology and Toxicology, School of Pharmacy, Shahid Beheshti University of Medical Sciences, Tehran, Iran; ^16^Department of Nutrition and Dietetics, Faculty of Pharmacy, and Centre for Healthy Living, University of Concepción, 4070386 Concepción, Chile; ^17^Faculty of Medicine, University of Porto, Alameda Prof. Hernâni Monteiro, 4200-319 Porto, Portugal; ^18^Institute for Research and Innovation in Health (i3S), University of Porto, 4200-135 Porto, Portugal; ^19^Institute of Research and Advanced Training in Health Sciences and Technologies (CESPU), Rua Central de Gandra, 1317, 4585-116 Gandra PRD, Portugal; ^20^Banat's University of Agricultural Sciences and Veterinary Medicine “King Michael I of Romania” from Timisoara, Timisoara, Romania; ^21^Victor Babes University of Medicine and Pharmacy of Timisoara, Department of Microbiology, Timisoara, Romania; ^22^Multidisciplinary Research Center on Antimicrobial Resistance, Timisoara, Romania; ^23^Preventive Medicine Study Center, Timisoara, Romania; ^24^Pharmaceutical Care Department, Ministry of National Guard-Health Affairs, Riyadh, Saudi Arabia; ^25^Department of Clinical Oncology, Queen Elizabeth Hospital, Kowloon, Hong Kong

## Abstract

Cardiovascular diseases (CVD) are one of the main causes of mortality in the world. The development of these diseases has a specific factor—alteration in blood platelet activation. It has been shown that phenolic compounds have antiplatelet aggregation abilities and a positive impact in the management of CVD, exerting prominent antioxidant, anti-inflammatory, antitumor, cardioprotective, antihyperglycemic, and antimicrobial effects. Thus, this review is intended to address the antiplatelet activity of phenolic compounds with special emphasis in preventing CVD, along with the mechanisms of action through which they are able to prevent and treat CVD. *In vitro* and *in vivo* studies have shown beneficial effects of phenolic compound-rich plant extracts and isolated compounds against CVD, despite that the scientific literature available on the antiplatelet aggregation ability of phenolic compounds *in vivo* is scarce. Thus, despite the current advances, further studies are needed to confirm the cardioprotective potential of phenolic compounds towards their use alone or in combination with conventional drugs for effective therapeutic interventions.

## 1. Introduction

Cardiovascular diseases (CVDs) are a major cause of human mortality and morbidity in Western countries, and they are considered a huge problem for todays' health care system [1]. Parallel to the improvement of life expectancy, deaths caused by CVDs have been increasing. Currently, smoking, obesity, poor nutrition, and sedentary lifestyles comprise the main contributors [2]. Scientists estimate that by 2030, in low-income countries, the number of individuals dying from CVD will be significantly higher in comparison to other infectious diseases and nutritional disorders combined [3].

**Figure 1:**
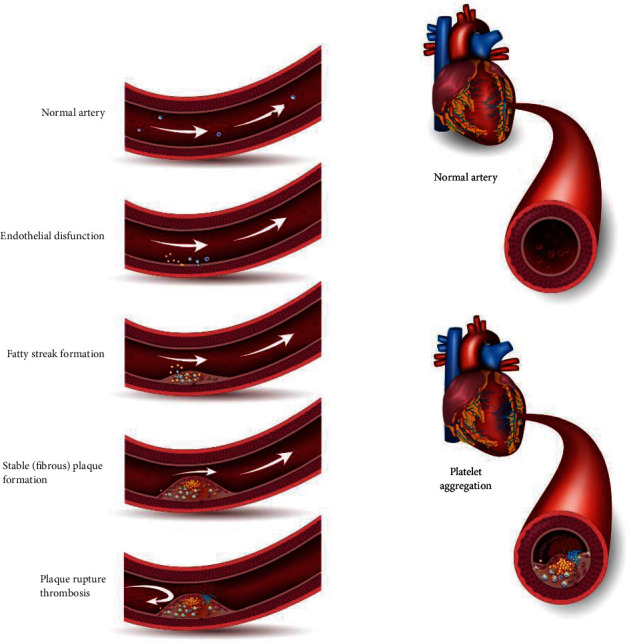
Process of platelet aggregation and formation of thrombosis during CVDs.

Changes in blood platelet activation are considered a specific key triggering factor for the development of CVD among others [4]. In healthy individuals, hemostatic plugs are formed as the blood clots at the bleeding site. In such situations, they are beneficial since they prevent both blood and plasma from escaping into surrounding tissue. On the other hand, thrombus expansion in the unruptured blood vessels can be harmful [5]. As a result of blood vessel injury and/or atherosclerotic plaque erosion, the endothelium liberates protein factors, enabling that way the platelets' adhesion to the subendothelium and initiating their activation. Activated platelets release biologically active ligands, including thromboxane A2 (TXA2), adenosine diphosphate, and serotonin, which further increase platelet activation, aggregation, and thrombus formation [6]. Thus, the proper control of platelet function is mandatory for the prevention of thrombotic events [7]. Indeed, platelet hyperactivity has been increasingly linked to the development and complication of certain CVDs **(**Figure 1**)**, including atherosclerosis, thrombosis, peripheral artery disease, myocardial infarction, and ischemic stroke [3].

On the other side, several side effects have been reported with the currently used synthetic antiplatelet agents (aspirin, clopidogrel, and dipyridamole). For example, the chronic consumption of aspirin implies an increased risk of developing intestinal ulcers and major bleeding [6]. Moreover, these drugs are also not completely effective in suppressing platelet aggregation besides increasing the risk of drug resistance and bleeding. As a consequence, these limitations have prompted researchers to launch the quest for natural alternatives as new, effective, and safer antiplatelet drugs. In a recent review, the use of natural products as therapeutic agents has been highlighted considering the data of the last four decades (Newman and Cragg [8]). The review highlighted the use of natural products as anticancer, antidiabetic, and multiple sclerosis agents. The polyphenolic compounds present in plant-based products have been found to play the central part of many scientific studies due to their potential health benefits, with special attention being given to their positive impact on CVD [9–11]. In fact, polyphenol-rich diets have revealed a great impact on the vascular system, improving both platelet and endothelial functions [12]. Moreover, platelet activation pathways involve arachidonic acid (AA), adenosine diphosphate, serotonin, and nitric oxide (NO) pathways. Numerous medicinal plants have also demonstrated their bioactivities in reducing platelet aggregation via these mechanisms [13]. Phenolic compounds may be better for treating CVD than synthetic antiplatelet agents, addressing the problems of drug resistance and bleeding that the synthetic antiplatelet agents cannot solve. A number of reviews which focused on the role of phenolic compounds in improving the cardiovascular health have been published recently [14, 15]. These reviews are more focused on the effect of the phenolic extracts on cardiovascular health, but limited aspects of antiplatelet aggregation activity of phenolic bioactive compounds in maintaining cardiovascular health were discussed. In this sense, considering the importance of the phenolic bioactive compounds in the management of CVDs by their antiplatelet activity, the aim of this review is to collate and summarize the finding of the researchers on polyphenolic compounds with antiplatelet activity, with emphasis on the possible molecular mechanisms responsible for their action against CVDs.

## 2. Basic Structure and Physicochemical Properties of Phenolic Compounds

Phenolic compounds account for about 40% of organic carbon circulating in the biosphere and are by definition any compound with a hydroxylated benzene ring. A polyphenolic compound has more than one phenol group, or more than one hydroxyl on a single benzene ring. Phenolic compounds come from simple, low-molecular-weight phenolic molecules to highly polymerized, high-molecular-weight, complex polyphenolic compounds [16]. They are synthesized either as soluble or cell-wall-bound compounds, generally appearing as esters and glycosides rather than free compounds. They are classified based on the number of phenolic rings that they contain and the radicals that bind the rings to one another [16]. The hydroxyl group of phenolics is influenced by the presence of the aromatic ring which makes the hydroxyl group's hydrogen labile and then makes them weak acids [16].

Water solubility increases with the number of hydroxyl groups. For analytical purposes, methanol, ethanol, water, and alcohol-water mixtures have been the most commonly used solvents to dissolve and extract phenolic compounds. The intense absorption in the spectrum of the UV region is exhibited by all phenolic compounds, and the colored ones also absorb strongly in the visible region [16].

Polyphenolic flavonoids constitute the largest group of low-molecular-weight phenolic compounds, characterized by a phenyl-benzo-pyran chemical structure consisting of two aromatic C6 rings (A and B) and a heterocyclic benzopyran ring (C) with one oxygen atom [16]. They are crystalline compounds; some are colored, while others are colorless. They occur as aglycones, glycosides, and methylated derivatives, with differences in glycosides being attributed to the number of positions for glycosylation, types and numbers of sugars involved, and level of glycosylation. Briefly, glycosides can either be O- or C-linked in positions 3 or 7 to L-rhamnose, D-glucose, glucorhamnose, galactose, or arabinose [16].

Phenolic acids (phenolcarboxylic acids) are members of the phenolic compounds' group containing a phenolic ring and at least one organic carboxylic acid function [16]. Tannins are water-soluble polyphenols, characterized by their relatively high molecular weight, being often found in complexes with alkaloids, polysaccharides, and proteins. Stilbenes exhibit a C6-C2-C6 structure derived from the same biosynthetic pathway as flavonoids and are featured by the presence of a 1,2-diphenylethylene nucleus with hydroxyl groups substituted on the aromatic rings [16].

### 2.1. Main Sources of Phenolic Compounds

According to Lattanzio [17], plant phenolic content depends on several factors, such as growing conditions, cultivation techniques, cultivar, and ripening process. In addition, while they are natural antioxidants and health promoting phytochemicals, phenolic compounds are among the health promoting phytochemicals found abundantly in plant-based foods, including fruits, vegetables, spices, and whole grains, especially cereals [17]. The subclass of flavonoids is widely addressed for their protective effects on CVDs by inhibiting platelet aggregation. Plant-based food groups are known to be richer in such compounds than others. Below, some few examples of plant-based food groups which are rich in these flavonoid compounds are presented. The concentrations of a particular flavonoid are given in mg/100 g edible portion.

- Fruits: berries (blueberries (flavones: 7.5–19.7), raspberries (flavonols: 1.11–1.114), strawberries (flavonols: 1.3–1.65), and cranberries (flavones: 0.03)), cherry (flavonols: 2.43–28.6), apple (flavones: 0.01–0.12; flavonols: 0.42–3.87; flavan-3-ols: 6.64–12.3; and anthocyanidins: 0–5), grape (flavonols: 1.05–2.39), lemon (flavonols: 1.67; flavanones: 49.8), lime (flavonols: 0.40), banana (flavonols: 0.18), orange (flavonols: 0.22–0.73; flavanones: 29.0–42.6), pears (flavan-3-ols: 1.88–4.81; anthocyanidins: 2.06), plums (flavonols: 0.90–12.5; anthocyanidins: 0.30–558.2), peaches (flavonols: 0.45–0.88; anthocyanidins: 0.97–1.92; and flavan-3-ols: 1.87–16.3), and apricot (flavan-3-ols: 8.41)
- Cereals and legumes: soybean (flavan-3-ols: 37.4), cowpea (flavonols: 21.9), sorghum (flavones: 2.99–6.47; flavanones: 1.96), broad beans (flavanols: 0.9), beans (anthocyanidins: 2.74–44.5; flavan-3-ols: 0.10–324.2), purple wheat (anthocyanidins: 25.9), and peanuts (flavan-3-ols: 0.66)
- Vegetables: eggplants (flavones: 0.03), onions (flavonols: 3.63–46.7; flavon-3-ols: 0–0.08; and anthocyanidins: 9.56), broccoli (flavonols: 1.05–11.2), carrots (flavonols: 0.49), cabbage (flavonols: 0.05–22.5), lettuce (flavonols: 1.63–7.63), and tomatoes (flavonols: 0.03–0.8)
- Herbs and spices: chili, coriander (flavonols: 52.9), garlic (flavonols: 3.61), ginger (flavonols: 0.19–33.6), turmeric (flavonols: 6.96), and thyme (flavones: 47.75)
- Beverages: red wine (flavones: 0.04–0.17), tea (flavan-3-ols: 9.8–324.2), coffee (flavan-3-ols: 0.08; flavonols: 0.10), and cocoa (flavan-3-ols: 1.33–52.7)

Therefore, there is a chance of increased consumption by human on a daily basis, since they are widespread in plant-based foods and are easily absorbed due to their simplicity.

## 3. Phenolic Compounds and Health Promotion

Phenolic compounds are highly abundant secondary metabolites in the plant kingdom, possessing aromatic rings with one or more hydroxyl substituents or some functional derivatives, such as esters, methyl ethers, and glycosides [18]. Both taste and color, as well as some features of vegetables and fruits are strongly associated with the presence of such compounds, widely recognized for their wide range of activities, making them extremely beneficial to human health.

The physiological functions of phenolic compounds are vast because of their beneficial effects, not only for health promotion and maintenance but also for therapeutic purposes, given their wide range of pharmacological activities; thus, taken together, these aspects have made phenolic compounds highly important secondary metabolites and the target of an intense investigation in current times [19]. Taking a look at their versatile health benefits, they are effective antioxidant, antitumor, antimicrobial, antihyperglycemic, immunomodulatory, cardioprotective, vasodilatory, antithrombotic, anti-inflammatory, and UV radiation skin protective agents, and therefore interesting candidates for pharmaceutical and medicinal applications [20–23].

Due to the medicinal plant's abundance in phenolic compounds, interesting biological activities have been reported, which are extremely useful in the prevention of the onset of age-related diseases and closely related to high oxidative stress levels. With regard to the broad-spectrum activities, there is a shift towards natural product industrialization, especially in pharmaceutical and cosmetic industries. For example, flavonoids regulate key proteins involved in inflammation and signal transduction pathways [24]. Both the absorption and metabolism of these dietary phenolic compounds determine the extent of their health benefits, which in turn are determined by their structure (conjugation with other phenolics, degree of glycosylation/acylation), solubility, and molecular size. Their effectiveness decreases with the substitution of the hydroxyl groups in their structure for sugars; thus, aglycones exhibit more potent activities than their corresponding glycosides.

## 4. Phenolic Compounds That Inhibit Platelet Aggregation via Affecting Vascular Environment

### 4.1. Phenolic Compounds and Cardiovascular Activity: Emphasis on Molecular Ways

The regular consumption of diets rich in fruits, vegetables, olive oil, and wine, composed of a wealth of phenolic compounds, exert a wide range of beneficial properties, such as antioxidant and anti-inflammatory effects as well as metabolic modulatory activities to hinder not only the disease onset but also its progression [25–28].

#### 4.1.1. Cardioprotective Activity of Phenolics

CVD pathogenesis has been increasingly linked to oxidative stress. Typically, featured by an inflammatory condition, CVD is recognized as one of the major causes of death worldwide, with a rise in prevalence in developed countries [29]. As polyphenols are known for their antioxidant, immunomodulatory, and vasodilatory properties, an increased intake of such dietary antioxidants contributes to CV risk reduction [9] and inhibits oxidation of human low-density lipoproteins (LDL), ultimately preventing atherosclerotic plaque formation. The mechanisms involved in the cardioprotective effects of phenolic compounds have been studied and reported in preclinical (*in vitro* and *in vivo*) and clinical studies, acting via inhibition of ROS production, mitochondrial dysfunction, apoptosis, nuclear factor kappa B (NF-*κ*B), p53, and DNA damage. For example, resveratrol as a case study, is a phenolic compound (stilbene) widely abundant in grapes and red wine that confers extraordinary cardioprotective effects by acutely improving endothelial function in coronary heart disease (CHD) patients [30].

**Figure 2:**
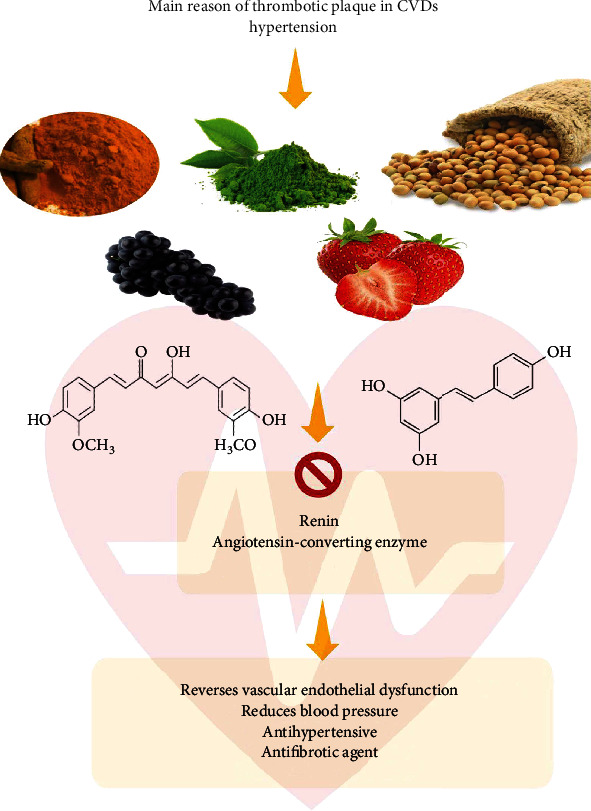
Antithrombotic and antihypertensive properties of the phenolic compounds.

Regarding CVD risk factors, hypertension is the most prominent one, contributing to one third of global mortality. Recently, naturally occurring phytochemicals have been employed not only to reduce but also to manage hypertension risk [31]. The renin-angiotensin-aldosterone (RAAS) system has been identified as an important target in the treatment/management of hypertension, myocardial infraction, stroke, and kidney diseases [32], given its role in the maintenance of vascular tone/tension. Briefly, the RAAS system mediates vascular tension via a sequential conversion of angiotensinogen to angiotensin II by a series of enzymatic cleavages. The angiotensinogen is cleaved by renin to produce angiotensin I, which is further cleaved to produce angiotensin II by the angiotensin-converting enzyme (ACE). Angiotensin II is a potent vasoconstrictor, and its presence also promotes aldosterone production, thus contributing to hypertension [32]. Hence, inhibitors of renin and ACE have shown to be beneficial in the treatment of vascular tension and as antifibrotics [33]. Recently, phenolic compounds have revealed promising effects both as ACE and renin inhibitors, with phenolic-rich foods also revealing good antihypertensive abilities in experimental models (Figure 2) [34, 35].

Experimental and clinical studies have suggested that flavonoids and flavanol-rich foods could reduce blood pressure and CVD risk in humans [36]. For instance, caffeic acid along with its 19 novel derivatives, chlorogenic acid, quercetin, and captopril showed prominent effects as inhibitors of ACE and renin, as well as modulators of aldosterone secretion [31]. Other evidences have also shown that phenolic compounds, particularly flavonoids, are able to reverse vascular endothelial dysfunction [37].

Indeed, phenolic supplementation has been shown to boost endothelial function by stimulating endothelium-derived NO bioactivity, and this may explain some of the favorable effects of high phenolic intake seen in epidemiological studies [37]. For example, a randomized controlled trial performed in pre- and hypertensive patients, revealed that olive oil enriched with its own phenolic compounds show more benefits on endothelial function than standard virgin olive oil [38].

### 4.2. Role of Phenolic Compounds as Antiplatelet Agent via Redox Modulation

As the largest phytochemical molecules grouped under phenolic compounds, phenolic acid and flavonoids are commonly known to have great antioxidant properties and prove to be more effective than vitamins C and E and carotenoids [39]. These antioxidant abilities are mediated through several mechanisms, such as scavenging free radicals, suppressing ROS formation, inhibiting some enzymes, chelating trace metals, and upregulating or protecting antioxidant defense to ensure a proper redox balance [40]. The hydrogen-donating specificity and interaction of their hydroxyl groups (acting as the antioxidant) with ROS is a termination reaction which breaks the cycle of a new radical generation. The main factor on antioxidant/reduction activity of phenolic compounds is the number and position of its hydroxyl groups which are strengthened by steric hindrance; thus, flavonoids possess more hydroxyl groups and higher antioxidant activity than the other molecules of the group [41, 42].

ROS are generated intracellularly and exogenously as a byproduct of normal metabolism or due to cells' exposure to some environmental triggers [43]. The imbalance between ROS generation and defense mechanism is known as oxidative stress, which is pivotal in CVD development [44, 45]. Oxidized LDL promotes vasoconstriction and progression of platelet aggregation by promoting the smooth muscle cells' proliferation and inhibiting the endothelial nitric oxide synthase (eNOS) [46].

**Figure 3:**
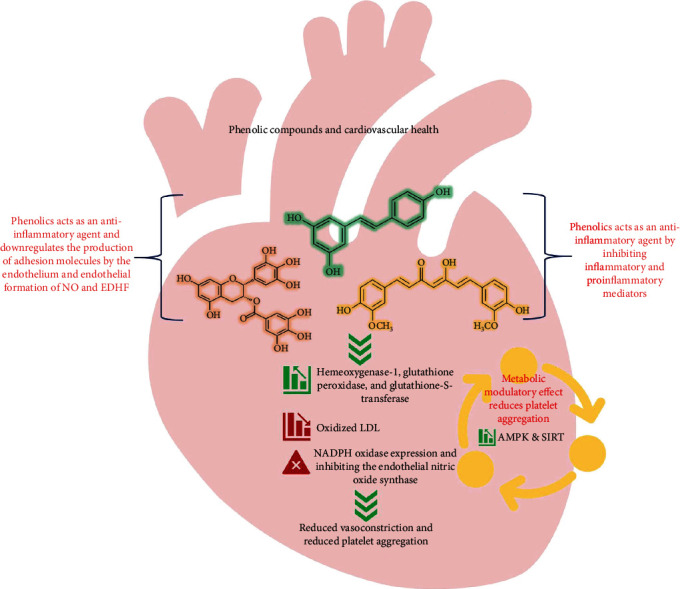
Role of phenolics as antiplatelet factors via redox modulation, anti-inflammatory responses, and metabolic modulatory effect.

Phenolic compounds exert an antioxidant activity via their free radical scavenging properties and inhibiting ROS-generating enzymes (e.g., iNOS), as well as boosting antioxidant enzyme activity, like hemeoxygenase-1, glutathione peroxidase, and glutathione-S-transferase in cardiac and aortic smooth muscle cells [44, 45]. They are also able to regulate vascular reactivity by inducing nuclear Nrf2 accumulation and targeting eNOS, thereby enhancing NO bioavailability [47, 48]. Polyphenols also contribute to the antioxidant defense of endothelial cells by reducing NADPH oxidase expression [49]. Several *in vitro* studies have reported that phenolic compounds could effectively reduce oxidized LDL and increase the level of high-density lipoproteins (HDL), ultimately improving endothelial function [50, 51]. Moreover, numerous phenolic compounds exert cardioprotective effects at a localized or systemic level by inducing antiplatelet effects [46], with such effects being majorly attributed to the *O*-dihydroxyl group in the A and/or B ring (Figure 3) [52].

### 4.3. Role of Phenolic Compounds as Antiplatelet Agent via Inflammatory Process

Production of proinflammatory mediators during inflammation is common in some cells, mainly in macrophages, with interleukins (IL), tumor necrosis factor- (TNF-) *α*, ROS, NO, and prostaglandins (PGs) being the most commonly produced inflammation mediators [53]. Also, the association of phenolic compound structures and their anti-inflammatory activity with different targets of inflammation have been established [54]. Indeed, they are able to modulate transcriptional factors, like downregulating NF-*κ*B or upregulating Nrf-2 [55, 56]. NF-*κ*B regulates the expression of several proinflammatory cytokines, such as IL-1*β*, TNF-*α*, and enzymes, like iNOS and cyclooxygenase (COX-2). Nrf-2 regulates the expression of anti-inflammatory enzymes by possessing an antioxidant responsive element (ARE) able to activate several antioxidant enzymes needed for redox balance. Thus, they inhibit the gene expression and the activity of proinflammatory mediators at the same time that they activate the expression and activity of anti-inflammatory mediators that are targets of transcription factors.

The inhibition of inflammatory mediators, such as ROS, NO, and PGE2, and proinflammatory mediators, like cytokines, TNF-*α*, and COX-2, is one of the major targets for CVD treatment (Figure 3). The overexpression of TNF-*α* and IL is linked to NF-*κ*B activation, which regulates the release of inflammatory mediators [57]. Additionally, phosphorylation of p38 mitogen-activated protein kinases (MAPK) plays an important role in chronic inflammation by activating NF-*κ*B as well as regulating the NO and proinflammatory gene production from macrophages [58, 59].

Procyanidins were found to reduce the protein expression of iNOS, COX-2, lipoxygenase- (LOX-) 15 and some proinflammatory cytokines, such as IL-1*β*, TNF-*α*, and monocyte chemoattractant protein- (MCP-) 1 [58, 60, 61]. These effects might be due to suppression of NF-*κ*B activity via downregulation of p38 and MAPK pathways [44, 58]. Rius et al. [62] proved that resveratrol supplementation may partially protect against CVD especially during the early atherosclerotic phase. This effect could be due to a decrease in the overexpression of intercellular and vascular cell adhesion molecules by inhibiting the NF-*κ*B pathway in TNF-*α*-activated endothelial cells [63], and other reports have shown that it is related to a reduction of circulating levels of MCP-1 and MIP-1*α* [62]. Polyphenols extracted from roasted cocoa beans suppressed inflammation via oxidative pathways, which lead to an increase in oxygen consumption by mitochondria and ATP production via oxidative phosphorylation [64].

As stated above, phenolic compounds present in extravirgin olive oil have also been stated to be potent anti-inflammatory agents by preventing the expression of iNOS, COX-2, LOX, and phospholipase A2 and thus blocking the production of eicosanoids (PGI2, leukotriene B4) [65]. They can also reduce platelet aggregation by decreasing the production of thromboxane B2 (TXB2) and 27-hydroxyleicosatetraenoate [66]. The same findings have also been said about resveratrol, which forms stable complexes in platelet COX-1 channels [67]. Moreover, the supplementation with curcumin or cinnamon bark extract was able to reduce the levels of C-reactive protein (CRP), an acute phase protein that plays a key role in CVD progression [68, 69]. The potential therapeutic and health-promoting roles of curcumin are also highlighted in the recent review by Moballegh Nasery et al. [70].

On the other hand, endothelial dysfunction results in the formation of vasoconstrictive factors, such as endothelin-1 in the arterial wall, implicated in CVD development [71]. Dietary polyphenols have also been shown to downregulate the production of adhesion molecules by the endothelium and to modify the endothelial formation of NO and endothelium-derived hyperpolarizing factor (EDHF), which improve endothelial function [9].

### 4.4. Role of Phenolic Compounds as Antiplatelet Agents via Metabolism Modulation

CVDs are related to alterations in metabolism. Dasgupta and Milbrandt in 2007 found that resveratrol can target and activate AMP-activated protein kinase (AMPK), having an important role in reducing fat accumulation, cholesterol synthesis, and inflammatory cytokines [72]. Resveratrol could also stimulate sirtuin 1 (SIRT1) at an amplitude of ~10-fold, which is a NAD-dependent lysine deacetylase that plays a vital role in energy metabolism **(**Figure 3) [73]. SIRT1 is known to regulate a variety of cell functions, mainly mitochondrial function, by activating the transcriptional activity of peroxisome proliferator-activated receptor-gamma coactivator- (PGC-) 1*α* and triggering the activation of AMPK [74, 75]. Stimulation of SIRT1 and AMPK boosts the eNOS activity in human coronary arterial endothelial cells and increases NO production and mitochondrial biogenesis, which triggers vasodilation and decreases atherosclerosis [76]. Recent data have shown that polyphenols interact directly with the activator site of both estrogen receptor- (ER-) *α* and ER-*β*, leading to eNOS activation and stimulation of NO production and endothelium-dependent vasorelaxation [77, 78].

## 5. Phenolic Compounds with *In Vitro* Antiplatelet Aggregation Activity

The inhibition of platelet aggregation and thrombus formation comprises a vital target in preventing atherosclerotic events [79]. This concept has motivated researches to find therapeutic strategies targeting to reduce platelet aggregation. In fact, diets rich in phenolic compounds could represent a natural alternative for inhibiting platelet aggregation in a dose-dependent manner and helping reduce the individual risk of developing CVD [80]. From a mechanistic point of view, the inhibitory activity of phenolic compounds greatly depends on the phenolic class and is mainly due to their anti-inflammatory and antioxidant capacities, with an IC_50_ in the range of *μ*M [81]. Pignatelli et al. [82] have reported that a combination of 25 *μ*M catechin/L and 5 *μ*M quercetin/L can synergistically inhibit platelet aggregation by blunting hydrogen peroxide production. Wang et al. [83] reported that kaempferol inhibits NADPH oxidase, thus reducing the ROS production. Similarly, Meshkini and Tahmasbi reported in 2017 that the antiplatelet activity of walnut hull extract is linked to its capacity to inhibit the rise in ROS levels induced by thrombin in platelets [7]. Cocoa polyphenols reduced platelet NADPH oxidase activation and the platelet formation of ROS and eicosanoids [84].

The main reported mechanisms of action for phenolic compounds, other than the antioxidant effects, include the suppression of cytoplasmic Ca^2+^ increase and inhibition of thromboxane formation and AA pathway [81, 85]. Quercetin was found to completely inhibit AA-induced platelet aggregation at 200 *μ*M [86] and to inhibit platelet aggregation induced by thrombin by impairing Ca^2+^ mobilization and serotonin secretion [87]. Resveratrol could also inhibit the arachidonate-dependent synthesis of inflammatory agents, such as TXB2, hydroxyheptadecatrienoate, and 12-hydroxyeicosatetraenoate [67]. Apart from the antiplatelet activity of resveratrol, it also modulates the expression of noncoding RNAs in ovarian cancer cells (Vallino et al., 2020). Another study also showed the anticancer activity of resveratrol by inhibiting the STAT3 signaling pathway (Baek et al., 2016). Similarly, resveratrol might improve cardiovascular health by affecting the gene expression of platelet aggregating factors. Son et al. [88] reported that green tea catechins exert antithrombotic effects through the inhibition of TXA2 formation by modulating AA liberation and TXA2 synthase. Rutin and *α*-naphthoflavone have also been shown to inhibit phosphoinositide breakdown and several other steps, such as Ca^2+^ mobilization, protein kinase C (PKC) activation, and TXA2 formation in collagen-activated platelets [89, 90].

Flavonoids also inhibit the platelets' stimulation through phosphoinositide 3-kinase (PI3K)/PKB (AKT) and by extracellular signal-regulated kinase (ERK) 1/2, p38, and cJun N-terminal kinase (JNK) 1/2 pathways [91]. Several researches proved that quercetin at 50-60 *μ*M completely inhibits all PI3k isoforms [92]. Moreover, some flavonoids have been shown to inhibit phospholipase C, platelet-activating factor, or collagen-receptor antagonism and glycoprotein IIb-IIIa activation [93]. It has also been revealed that quercetin and catechin are able to downregulate the expression of GPIIb/IIIa in platelets by increasing the NO production [94]. Hydroxytyrosol acetate and hydroxytyrosol were also found to synergistically inhibit collagen-induced platelet aggregation [95]. The inhibition of phospholipases, tyrosine kinases, phosphodiesterases, LOX, and COX are other mechanisms involved [96]. *In silico* docking studies showed that resveratrol could form stable complexes in platelet COX-1 channels [67], while Hubbard et al. [97] concluded that quercetin inhibits collagen-induced phosphorylation mainly due to its tyrosine-kinase inhibitory activity.

**Figure 4:**
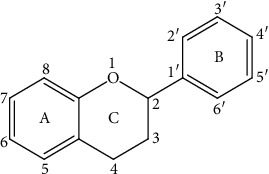
Basic structure of flavonoids.

From the point of view of a structure-activity relationship (Figure 4), the hydroxyflavones were more effective than their corresponding methoxyflavones, considering that the hydroxyl group position also influences platelet function [98]. This is explained by the fact that methylation changes the electrical charge of the flavonoid and so decreases its affinity for TXA2 receptors. Glycosylation also decreases the antiplatelet activity of flavonoids by enlarging their size, thus complicating binding to the receptor [85]. The double bond in C2–C3 and/or 4-C=O in the C-ring of flavonoids has also a key importance for the antiplatelet activities [99]. It was also observed that not only does the phenyl group of a B ring play a critical role in antiplatelet activity, but the heteroatoms of the B ring also largely influence this activity [98].

Data have also shown that apigenin, genistein, and luteolin have high affinity to the TXA2 receptor due to their structural characteristic conjugation, with the presence of a lactone structure [85]. Indeed, epigallocatechin gallate, catechin gallate, and epicatechin gallate are catechins containing a galloyl group in the 3′ position, all inhibiting thrombin-induced aggregation and phosphorylation of p38 MAPK and ERK1/2. Catechins without a galloyl group (catechin, epicatechin) or with a galloyl group in the 2′ position (epigallocatechin) did not inhibit platelet aggregation [100].

## 6. Phenolic Compounds with *In Vivo* Antiplatelet Aggregation Activity

*In vitro* evidence cannot be fully translated to the *in vivo* condition in animals because parent molecule administration is followed by the presence of conjugated metabolites in the plasma with lower biological effects [101]. Additionally, experimental data related to the antiplatelet aggregation activity of dietary polyphenols *in vivo* are scarce, and results are often conflicting. For example, Ostertag et al. [102] demonstrated in an ex vivo study that phenolic compounds affect the collagen-induced platelet aggregation and thrombin receptor-activating peptide-induced P-selectin expression but only at very high nonphysiologically attainable concentrations. In streptozotocin-induced diabetic rats, Schmatz et al. [103] also proved in an *ex vivo* study that moderate consumption of grape juice and red wine modulates the hydrolysis of the adenine nucleotides and decreases platelet aggregation.

Schumacher et al. [104] studied the effect of 300 mL chicory coffee rich in caffeic acid given daily to 27 healthy volunteers for 1 week. They found that the whole blood and plasma viscosity were both significantly decreased. In another study, 20 healthy subjects daily consumed 7 mL/kg of both red wine and purple grape juice for 14 days [104]. Platelet aggregation was inhibited and platelet-derived NO production increased, whereas the superoxide release decreased significantly [105]. In an intervention trial that lasts for 8 weeks, berry consumption resulted in favorable changes in platelet function, and antiplatelet activity was induced by both ADP and collagen [106]. Similar findings were also stated when consuming 2 or 3 kiwi fruits per day for 28 days [107]. The daily consumption of 50 mL of pomegranate juice (1.5 mmol total polyphenols) for 2 weeks was found to reduce LDL susceptibility to aggregation and retention and to increase the activity of serum paraoxonase by 20% [108]. Data from several studies shows that the consumption of 100 mg flavanols (equivalent to 11 g dark chocolate, 52 g milk chocolate, or 50 to 100 mL cocoa drink) also inhibits the collagen–epinephrine- and collagen–ADP-induced closure [109]. Hamed et al. [110] proved that the intake of dark chocolate (700 mg flavonoids/day) for a week significantly reduced ADP- and AA-induced platelet activation in addition to activated glycoprotein IIb/IIIa. Similar findings were reported by Rull et al. [111] who proved that dark chocolate (with high and low flavanol levels) supplementation for 6 weeks lowered platelet responsiveness to ADP and to thrombin receptor activator peptide. Furthermore, Wright et al. [112] found that both methylated and sulphated flavonoid metabolites have higher platelet inhibitory effects than the glucoronidated metabolites.

Conversely, in a study conducted on 20 subjects, a polyphenol-rich meal every lunchtime for 5 days had no ex vivo effect on platelet aggregation although the total plasma flavonoids significantly increased [113]. In the same way, a daily supplementation with 200 mg of flavonoids from grape seeds had no effect on platelet aggregation in a double-blind randomized study performed in male smokers [114]. Platelet aggregation did not evidence significant differences in 21 postmenopausal women supplemented with wine polyphenols [115]. Additionally, the daily intake of 2 g of cocoa flavonols in healthy volunteers during 12 weeks did not exert significant differences in platelet aggregation [116].

Taken together, these findings underline that further *in vivo* studies are absolutely required to confirm the potentiality of phenolic compounds as antiplatelet aggregation agents. This potentiality renders polyphenols an important dietary element in the prevention of CVD and possibly an alternative to pharmacological treatments of platelet aggregation.

## 7. From Effects of Phenolic Compounds to Absorption and Bioavailability

**Figure 5:**
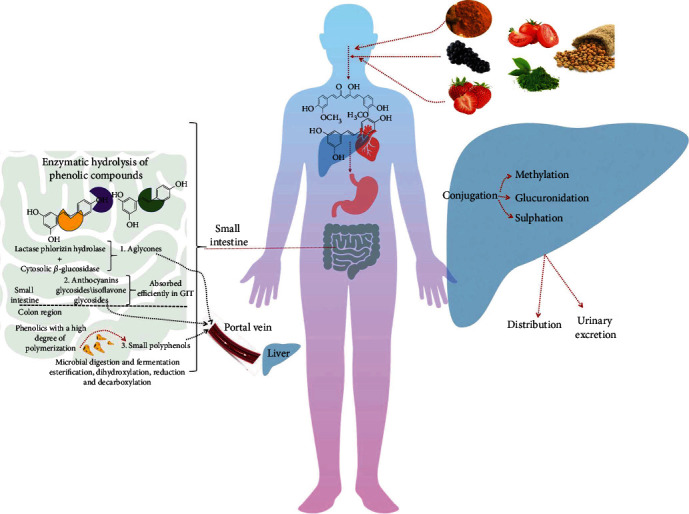
Absorption and bioavailability of phenolics as an antiplatelet agent through the liver distribution system.

The bioavailability of polyphenols and related metabolites following oral intake has been viewed as a hot research topic in the last decades. Indeed, results obtained so far have indicated that the bioavailability of phenolic compounds is related to the cleavage and release of the aglycone by digestive enzymes and microbial fermentation, as shown in Figure 5 [117]. The identification of phenolic compounds in biological samples to check its bioavailability, metabolism, and intestinal absorption is very crucial. These metabolomic analyses are frequently done using nuclear magnetic resonance and mass spectrometry ([118]).

Lactase phlorizin hydrolase and cytosolic *β*-glucosidase were found to be the main digestive enzymes that hydrolyze phenolic glycosides and release the aglycones which enter the epithelial cells [119, 120]. Before entering into the systemic circulation, polyphenolic aglycones undergo some degrees of phase II metabolism through the action of sulfotransferases, uridine-5′-diphosphate glucuronosyl transferases, or catechol-*O*-methyltransferases [121]. On the other side, compounds with a high degree of polymerization are exposed to microbial catabolism in the colon before reaching the liver, where they can also be subjected to conjugation [122]. Also worthy of note is that the different compositions of the colonic microflora between individuals lead to variations in the released metabolites [123]. In contrast, some polyphenols show specific pharmacokinetic features, as their glycosides are considered to be more bioavailable than their aglycones. As an example, some anthocyanins, glycosides, and isoflavone-glycosides could be efficiently absorbed across the gastrointestinal mucosa [124, 125]. For instance, Kay et al. [126] revealed that the absorption rate for quercetin glycosides is higher than that of the aglycones.

## 8. Phenolic Compounds: Looking at Adverse Effects, Drug Interaction, and Related Toxicity

The beneficial effects of phenolic compounds could be diminished by aspects as lifestyle, gender, age, genetic factors, underlying diseases, and interactions with conventional drugs, the latter being of great importance with regard to safety concerns. In fact, although the risk of adverse effects due to interactions between herbal medicinal products and conventional drugs is still an underexploited problem, some studies highlighting concerns regarding antithrombotic agents have been reported [96, 127, 128]. Overall, it is clear that the majority of these works do not address a single/specific drug and, on the contrary, preferentially consider anticoagulant (e.g., warfarin) and antiplatelet (e.g., aspirin, clopidogrel, or ticlopidine) drugs [129] as the whole focus of the study (Table 1).

The most referred mechanisms in such interactions include inhibition of thromboxane synthesis and/or COX activity and interference in drug metabolism, which globally results in synergistic effects with drugs, and ultimately, promoting platelet inhibition and increasing the risk of bleeding in some patients [127, 129]. Yet, in most cases, the mechanism of action is not deeply determined, and a great number of aspects on herbal-antiplatelet interactions remain unexplored. In addition, most of them lack the identification of the active compounds' class in such interactions, just referring to the name of the herbal product [130, 131]. Nevertheless, phenolic compounds of some herbal plants, such as *Camellia sinensis* (L.) Kuntze, *Citrus paradisi* Macfad., *Crataegus* spp., *Curcuma longa* L., *Crocus sativus* L., *Foeniculum vulgare* Mill. *Ginkgo biloba* L., *Matricaria recutita* L., and *Vaccinium myrtillus* L. have been pointed out as probable plant components capable of interacting with antithrombotic drugs.

## 9. Paving the Way for Effective Clinical Applications

Phenolic compounds are gaining a huge importance in the promotion, prevention, and maintenance of good health, as well as in the treatment of multiple diseases, because of their remarkable bioactive effects, with special emphasis on their antioxidant, antitumor, antihyperglycemic, cardioprotective, antithrombotic, vasodilatory, and anti-inflammatory activities. This wide range of activities makes such biomolecules of hot potential for both pharmaceutical and medical applications.

As stated, phenolic compounds are able to modulate some transcriptional factors (e.g., NF-*κ*B) to regulate the expression of some proinflammatory cytokines (e.g., IL-1*β* and TNF-*α*) and even enzymes (e.g., iNOS and COX-2) that are present in inflammatory processes. NF-*κ*B is associated with the Nrf-2 regulation that regulates the expression of anti-inflammatory enzymes [55, 56]. Considering such mechanisms and their ability to inhibit ROS, the cardioprotective effects of such molecules are due to its ability to improve endothelial dysfunction in CHD [37], since it also increases the bioactivity of endothelial NO. Moreover, evidence has stated that flavonoids reverse endothelial dysfunction in addition to lowering blood pressure [36].

To name a few mechanisms, there is increasing *in vitro* evidence that shows that polyphenols reduce platelet NADPH oxidase activation [83, 84], ROS formation, thromboxane formation, AA pathway [84, 85], and platelet stimulation through PI3K/AKT [91]. Nonetheless, there is still little *in vivo* evidence showing that polyphenols can inhibit platelet aggregation, increase NO production [105], inhibit ADP-induced platelet activity [106], and reduce AA-induced platelet activation [110]. So, there is no doubt that the *in vitro* data cannot be translated into the *in vivo* condition, because the molecules undergo biotransformation reactions that alter their bioavailability. As a consequence, this would lead to discrete and contradictory biological effects.

There is increasing evidence showing that the effectiveness of selective phenolic compounds is supported by their important role in whole foods, where when used in combination, such as through the intake of whole plant foods with a mixture of polyphenols, better antiplatelet effects are reached when compared to isolated compounds. For these reasons, further studies are needed to confirm its potential.

## 10. Conclusions and Upcoming Perspectives

Phenolic compounds have revealed several positive effects in CVD models *in vitro* and *in vivo*. However, for *in vivo* models, the evidence is scarce. However, with the described evidence, it can be affirmed that polyphenols are a key element in CVD prevention. Regarding the effectiveness of phenolic compounds, little has been studied about their interactions with medications, like anticoagulants and antiplatelets. Such interactions, that include mechanism of inhibition of the synthesis of thromboxane or COX activity, may increase the risk of bleeding in such patients, despite that there is a wide range of herbal products that interact with antithrombotic drugs in a synergistic manner.

Taken together, data presented here clearly underline the need for more *in vivo* studies and clinical trials to evaluate the phenolic compounds' potential and to guarantee their efficacy. Also, more investigations are desired on isolated or mixed phenolic compounds and their relationship with CVDs in order to elucidate their potential and success for both prevention and treatment. Finally, and not least important, as it is poorly described, is the design of more clinical trials, because most of the currently performed clinical studies with these herbal products lack identification and quantification of phenolic compounds.

## Figures and Tables

**Table 1:** Botanical species identified in literature acting as antithrombotic agents.

Species/herbal product Common name	Conventional drugs	Mechanism of action	Negative effect	Observations	References
*Allium sativum* Garlic	Anticoagulants and antiplatelets (aspirin, warfarin)	↔ thromboxane synthesis ↑ INR ↓ platelet aggregation	↔ with platelet ↓ blood clotting time Spontaneous spinal epidural hematoma causing paraplegia secondary	Ajoene, allicin, aliin, and thiocyanates are potentially responsible for the interaction Need more clinical assays	([128]; [131]; [132]; [133]; [134])
*Borago officinalis* Borage seed oil	Anticoagulants and antiplatelets	Potentiate the effects of warfarin	↑ the risk of bleeding		[131]
*Camellia sinensis* Green tea	Anticoagulants and antiplatelets	⦸ platelet aggregation ↔ coagulation mechanisms (e.g., antagonized the effects of warfarin)		Catechins, tannins, and others (caffeine, vitamin k) are potentially responsible for the interaction *In vitro* studies Clinical significance unknown	([127]; [135])
*Carthamus tinctorius* Safflower	Anticoagulants and antiplatelets	Synergism	↑ the effects of blood thinning agents		[131]
*Cnicus benedictus* Blessed thistle	Anticoagulant and antiplatelets	Synergism	↑ bleeding risk	Theoretical interaction	[127]
*Commifora mukul* Guggul	Anticoagulant and antiplatelets	Synergism	↑ bleeding risk	Theoretical interaction	[127]
*Citrus paradise* Grapefruit, juice	Cilostazol, clopidogrel, and ticagrelor	Synergism (cilostazol, ticagrelor) ⦸ CYP2C19 and CYP3A4 (clopidogrel) ⦸ CYP3A4-mediated first pass metabolism (ticagrelor)	↑ antiplatelet effects (cilostazol, ticagrelor) ⦸ effect (clopidogrel) Purpura (cilostazol)	Coumarins and flavonoids are potentially responsible for the interaction Need more clinical assays	([131]; [136]; [137]; [138])
*Crataegus monogyna*, *Crataegus laevigata*, *Crataegus* spp., *Crataegus oxyacantha* Hawthorn	Anticoagulants and antiplatelets	⦸ biosynthesis of thromboxane A2	Potentially ↑ risk of bleeding	Procyanidins, phenolic acids, and flavonoids are potential responsible for the interaction Need more clinical assays	([127]; [134]; [139])
*Curcuma longa* Turmeric	Antiplatelets	Synergism	↑ risk of bleeding	Curcumin and *ar*-turmerone are potentially responsible for the interaction	([127]; [131]; [140])
*Cassia senna* Senna	Anticoagulant and antiplatelets	Synergism	↑ risk of bleeding		[127]
*Crocus sativus* Saffron		Synergism ↓ platelet aggregation		4-*trans*-Crocin, picrocrocin, safranal, and flavonoids are potentially responsible for the interaction	[127]
*Eugenia aromatic* Clove	Anticoagulants and antiplatelets	Synergism ↓platelet aggregation *in vivo* and in clinical studies ↑ INR	↑ risk of bleeding		[131]
*Foeniculum vulgare* Fennel	Antiplatelets (aspirin)	Synergism	↑ risk of bleeding	Anetol, fenchone, isorhamnetin, luteolin, and naringenin are potentially responsible for the interaction	[141]
*Ganoderma lucidum* Reishi mushroom	Anticoagulant and antiplatelets	Synergism	↑ risk of bleeding	Theoretical interaction	[127]
*Ginkgo biloba* Ginkgo	Anticoagulants (warfarin and others), antiplatelets (aspirin, clopidogrel), NSAIDs	⦸ platelet aggregation ⦸ platelet-activating factor and altering bleeding times ↑ anticoagulant effect of warfarin and aspirin Platelet-activating factor receptor antagonists	↑ risk of bleeding Severe spontaneous bleeding Subdural haematomas	Ginkgolides (ginkgolide B), bilobalides, flavonoids, terpenic lactones, amentoflavone, and ginkgolic acids are potentially responsible for interaction Need more clinical assays	[127, 128] ([132]; [133]; [134]; [135]; [142])
*Glycyrrhiza glabra* Licorice	Anticoagulants and antiplatelets	⦸ thrombin ⦸ platelet aggregation			[134]
*Harpagophytum procumbens* Devil's claw	Anticoagulants (e.g., warfarin) and antiplatelets	Synergism	Purpura (warfarin)	Need clinical assays	([127]; [131]; [143])
*Lavandula angustifolia* Miller lavender	Anticoagulant and antiplatelets	Synergism			[127]
*Larrea tridentate*, *Larrea divaricate* Chaparral	Aspirin	↓ platelet aggregation	Delayed its onset	Nordihydroguaiaretic acid (NDGA) are potentially responsible for the interaction	([127]; [131])
*Leonurus cardiaca* Motherwort		↓ platelet aggregation ↓ fibrinogen levels			[134]
*Matricaria recutita* Chamomile	Anticoagulants and antiplatelets	Synergism		Coumaric compounds are potentially responsible for the interaction Need clinical assays	[144]
*Monascus purpureus* Red yeast rice	Anticoagulants (warfarin) and antiplatelets	↓ serum thromboxane concentration		Inconclusive conclusions	[131]
*Piper methysticum* Kava	Antiplatelets	⦸ thromboxane synthesis ⦸ cyclooxygenase		Isolated use is associated to reduced platelet size	[127]
Policosanol	Anticoagulants and antiplatelets	⦸ platelet aggregation *in vitro*			[131]
*Pinus pinaster* spp. *Atlantica* Pycnogenol®	Aspirin	↓ coagulation	↓ efficacy of acetylsalicylic acid		[131]
*Panax ginseng* Ginseng	Anticoagulants (warfarin), antiplatelets (aspirin) and NSAIDs	⦸ platelet aggregation ↓ platelet adhesiveness ↓ INR	↑ action of NSAIDs		([127]; [131]; [141])
*Pelargonium sidoides* Umckaloabo	Anticoagulant and antiplatelets	Synergism	↑ risk of bleeding	Theoretical interaction	[127]
*Rosmarinus officinalis* Rosemary	Antiplatelets	Synergism ⦸ platelets aggregation ↓ fibronectin ↓ fibrin			[127]
*Salvia hispanica* Chia	Anticoagulant and antiplatelets	Synergism	↑ risk of bleeding		[127]
*Salvia miltiorrhiza* Danshen	Anticoagulant and antiplatelets	↓ elimination of warfarin ⦸ cyclic adenosine monophosphate phosphodiesterase ↑ prothrombin time	↑ INR Over anticoagulation and antiplatelet effects: increased bleeding		([134]; [141])
*Serenoa repens* Saw palmetto	Anticoagulant and antiplatelets (aspirin)	Synergism ⦸ lipoxygenase ⦸ cyclooxygenase	↑ risk of bleeding		([127]; [131]; [141])
*Tanacetum parhenium* Feverfew	Anticoagulant (warfarin) and antiplatelets (aspirin)	Synergism	Additive platelet inhibition		([132]; [141]; [142])
*Trigonella foenum-graecum* Fenugreek	Anticoagulant and antiplatelets	⦸ effect	↑ INR		[127]
*Trifolium pratense* Red clover	Anticoagulant and antiplatelets	Synergism	Bleeding disorders		[127]
*Vaccinium myrtillus* Bilberry	Antiplatelets	Synergism		Anthocyanidins are potentially responsible for the interaction Theoretical interaction	[135]
*Zingiber officinale/*herbal products containing ginger Ginger	Anticoagulant (e.g., warfarin) and antiplatelets	NSAID-like activity (?) LOX ⦸ COX ↔ platelet		Gingerol, zingerone, and ginkgolide B are potentially responsible for the interaction Need more clinical assays	([128]; [135]; [144])

↑: increase; ↓: decrease; ⦸: inhibition; ↔: interference; INR: international normalized ratio; NSAIDs: nonsteroidal anti-inflammatory drugs.

## Data Availability

The data used to support the findings of this study are available from the corresponding author upon request.
